# A Systematic Review of Antidiabetic Mechanisms of *Passiflora edulis*: Beyond Glycemic Control

**DOI:** 10.1002/cbdv.71700

**Published:** 2026-10-20

**Authors:** Luiz Ricardo Teixeira de Farias, Mariáurea Matias Sarandy, Reggiani Vilela Gonçalves, Emanuella de Aragão Tavares, Silvana Maria Zucolotto

**Affiliations:** ^1^ Department of Pharmacy Federal University of Rio Grande do Norte Natal Rio Grande do Norte Brazil; ^2^ Department of Animal Sciences, Plants for Human Health Institute, NC Research Campus, NC State University Kannapolis North Carolina USA; ^3^ Department of General Biology Federal University of Viçosa Viçosa MG Brazil

**Keywords:** antidiabetic, diabetes mellitus, Passifloraceae, passion fruit

## Abstract

*Passiflora edulis*, commonly known as passion fruit, is traditionally used in various cultures for its calming and antidiabetic effects. This systematic review aimed to evaluate the potential of *P. edulis* on DM based on preclinical evidence from in vivo studies. This review was performed according to the PRISMA guide, using a structured search on the Embase, PubMed‐Medline, Web of Science, Cochrane and Scopus platforms. Twenty‐two studies met the inclusion criteria, and 18 reported antihyperglycemic effects of the plant, either alone or as an adjunct to insulin. Additional benefits included improvements in lipid and antioxidant profiles. The fruit was used in 16 studies, with the peel being the most frequent and effective part (11 studies), likely related to its high content of pectin, phenolics, and flavonoids. Proposed mechanisms include delayed intestinal glucose absorption, stimulation of insulin secretion, partial regeneration of pancreatic β‐cells, activation of the PI3K/AKT and AMPK pathways, and antioxidant and hypolipidemic actions. Despite these promising findings, significant methodological limitations, such as lack of dose and extraction process standardization and insufficient phytochemical characterization, affect reproducibility. Nevertheless, the data suggest that passion fruit peel, an often‐discarded agro‐industrial by‐product, may represent a promising source of bioactive compounds for diabetes research.

## Graphical abstract

*Passiflora edulis* demonstrates multitarget antidiabetic effects in preclinical models, particularly through peel‐derived preparations rich in pectin and flavonoids. The evidence indicates improvements in glycemic control, lipid metabolism, oxidative stress, and inflammation, involving pathways related to glucose absorption, insulin signaling, AMPK activation, and antioxidant defense, highlighting the therapeutic relevance of this agro‐industrial by‐product.

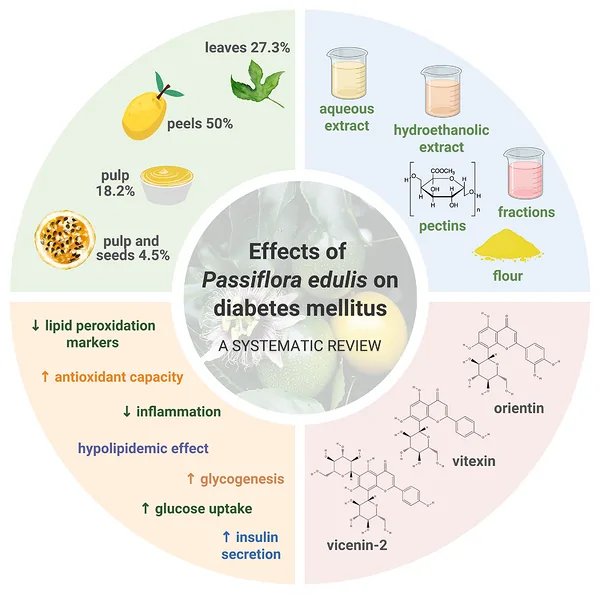

## 1 Introduction

Diabetes mellitus (DM) is considered a metabolic disorder that affects 1 in 10 people around the world [1]. It is characteristic of a group of heterogeneous disorders where the body does not produce insulin or does not use it properly, causing hyperglycemia. Excess blood glucose in the chronic form causes damage to target organs, such as the heart, blood vessels, eyes, kidneys, and nerves [2]. The use of commercial drugs; however, has some limitations, such as harmful side effects [3]. In this sense, research into natural products with antidiabetic pharmacological properties is of relevant interest for treating DM and its complications.

Among the different natural sources with antidiabetic activity, we highlight *Passiflora edulis*, also known as passion fruit. This plant is native but not endemic to Brazil and is distributed throughout tropical and subtropical areas of the globe. Its main variants are *Passiflora edulis* fo. *flavicarpa* O. Degener (yellow passion fruit), representing around 95% of worldwide passion fruit production (Carr, 2014). *Passiflora edulis* f. Sims (purple passion fruit) is commercialized on a smaller scale, along with other edible species of Passiflora [4]. However, these passion fruit variants exhibit important differences in their phytochemical and genetic profiles [5].

The widespread use of *P. edulis* in traditional medicine is due to its potential sedative, anxiolytic, diuretic, anthelmintic, antidiarrheal, among others [6]. Among its pharmacological properties, the antidiabetic potential has been widely reported in the literature, especially in preclinical studies [7, 8, 9]. Some studies also report the hypoglycemic properties of the phytoconstituents in passion fruit [10]. However, these studies using *P. edulis* for the treatment of DM vary in many aspects related to the plant material, such as the passion fruit variant used, the part of the plant, and the type of solvent and method of extraction, lack of phytochemical characterization, which compromises the reproducibility of studies and the reliability of the current evidence [6]. Furthermore, the type of mechanism involved in the antidiabetic action performed by passion fruit is not yet well known in these studies.

In this context, a study of the real influence of *P. edulis* on DM is necessary to understand the potential mechanisms involved, in addition to helping with research into natural products related to *P. edulis*, contributing to aspects that are often not highlighted in studies that seek to make relevant distinctions, such as the species used, parts used, types of extracts and their phytoconstituents involved [11]. In this scenario, a methodological literature analysis should be carried out to verify the potential of passion fruit (purple and yellow) on DM, and a systematic review can be used as an adequate, accurate, and reliable tool to evaluate state of the art about what has already been published in the literature.

## 2 Methods and Materials

### 2.1 Focus Question and Registration on the Prospero Platform

This systematic review was based on the following focus question: How does *P. edulis* (passion fruit) influence diabetes mellitus in vivo studies?

The research question was structured according to the PICO framework as follows: *Population (P)*: animal models of diabetes mellitus; I*ntervention (I)*: *P. edulis* and its derivatives; *Comparison (C)*: untreated or standard‐treated diabetic controls; *Outcomes (O)*: glycemic control, lipid profile, oxidative stress, and related metabolic parameters.

The registration number on the Prospero platform is CRD42023472406.

### 2.2 Search Strategy

This systematic review was performed according to the Preferred Reporting Items for Systematic Reviews and Meta‐Analysis (PRISMA) guidelines [12], which was used as a guide for the selection, screening, and eligibility of studies. To retrieve all relevant study registers, we used two parameters primarily. A structured and advanced search was performed across Embase, PubMed/MEDLINE, Web of Science, Scopus, and Cochrane library platforms on April 07, 2026, at 11 am. Following the initial database search, a manual screening of the reference lists of all relevant primary studies was conducted to identify additional eligible articles that may not have been captured in the initial query. No publication date or language restrictions were applied during the search process. The search strategy was based on two core domains: (i) Intervention: referring to the plant species *P. edulis*; and (ii) Biological process: focused on the context of diabetes mellitus disease.

The first search filter was built for the PubMed‐Medline database, according to the hierarchical distribution of MeSH (Medical Subject Headings) terms (www.ncbi.nlm.nih.gov/mesh, accessed on April 07, 2026) to reach all indexed studies. Furthermore, MeSH descriptors were combined with the TIAB (Title and Abstract) algorithm to get those studies not yet indexed. This filter used for the PubMed‐Medline platform was adapted for the Embase, Web of Science, Scopus, and Cochrane databases using the search algorithms TITLE‐ABS‐KEY or TS=. Additionally, no language limit was applied in the primary search. The complete search strategy is presented in Table S1.

Two independent reviewers searched the literature, removed duplicates, and examined titles and abstracts according to the eligibility criteria. In cases of disagreement, a third reviewer determined whether the study met the inclusion criteria.

### 2.3 Eligibility Criteria

After retrieving all relevant studies and removing duplicate studies, we evaluated the studies based on the inclusion and exclusion criteria. The included studies met the following inclusion criteria: (a) Studies that investigated the effects of any part of *P. edulis* on diabetes mellitus; (b) in vivo preclinical studies. Studies that met the following exclusion criteria were removed: (i) Studies that did not use *P. edulis* as an intervention; (ii) studies that did not investigate Diabetes mellitus; (iii) in vitro and/or ex vivo studies; (iv) clinical studies; and (v) secondary studies (i.e., editorials, commentaries, letters to the editor, reviews). When it was difficult to obtain the full‐text papers, the authors were requested to provide them by email.

### 2.4 Data Extraction and Synthesis From In Vivo Studies

After a detailed analysis of the articles that passed the eligibility criteria, the fundamental data from each study were extracted into tables structured according to the following categories: (i) Characteristics of the publications (authors, year of publication, country of origin, name, and journal impact factor); (ii) characteristics of the intervention species (species variant, part of the plant used, type of extraction, solvent used for extraction); (iii) characteristics of the experimental model (species, sex, age, weight, induction of diabetes mellitus); (iv) information about treatment with *P. edulis* (dose, route of administration, administration time); and (v) outcomes (*P. edulis* results, adverse effects, mechanisms). The data were extracted by two independent reviewers, and in cases of disagreement, a third reviewer resolved discrepancies and determined the final inclusion decision.

Due to the high heterogeneity among the included studies, particularly regarding experimental models, plant materials, extraction methods, dosing regimens, and outcome measures, a quantitative meta‐analysis was considered inappropriate and was therefore not performed.

### 2.5 Risk of Bias Assessment From In Vivo Studies

The risk of bias was analyzed using the Systematic Review Centre for Laboratory Animal Experimentation (SYRCLE) tool. This analysis is based on the Cochrane Collaboration tools for assessing risk of bias and adjusted for biases of particular interest in animal intervention studies. To improve the reliability and applicability of the analysis, predefined questions were used as a standard for the evaluation of researchers, based on the following domains: (i) Random sequence generation; (ii) allocation concealment; (iii) baseline characteristics; (iv) blinding of participants; (v) incomplete data; (vi) selective reporting; (vii) extract preparation; (viii) phytochemical characterization; (ix) evaluation of isolated compound; (x) ethical approval; (xi) statistical methods; and (xii) other bias. For each study, the items were scored as “low risk of bias”; “high risk of bias”; or “unclear”. Data assessed through SYRCLE were expressed graphically using the Review Manager 5.3 program (The Nordic Cochrane Centre, Copenhagen, Denmark, The Cochrane Collaboration).

## 3 Results

### 3.1 Prisma Guidelines

After searching the Embase, PubMed‐Medline, Web of Science, Scopus, and Cochrane databases, 414 studies were found. Of these, 228 were duplicates. One hundred and eighty six studies underwent an initial screening to remove those that were not experimental studies or primary research by reading the titles and abstracts. After this stage, 111 studies were retrieved to read the full text and apply eligibility criteria. Of the studies removed at this stage, 15 were clinical studies, 23 were different disease studies, 50 were different species studies, and 3 were in vitro and/or ex vivo studies. Twenty studies were included in the systematic review. The reference lists of all included studies were analyzed to ensure the identification and addition of relevant studies not found in the databases. We found four relevant records. However, only 2 were retrieved and included, thus totaling 22 articles (Table S2). Figure 1 presents the PRISMA 2020 flowchart highlighting all the steps taken to obtain all relevant studies.

**FIGURE 1:**
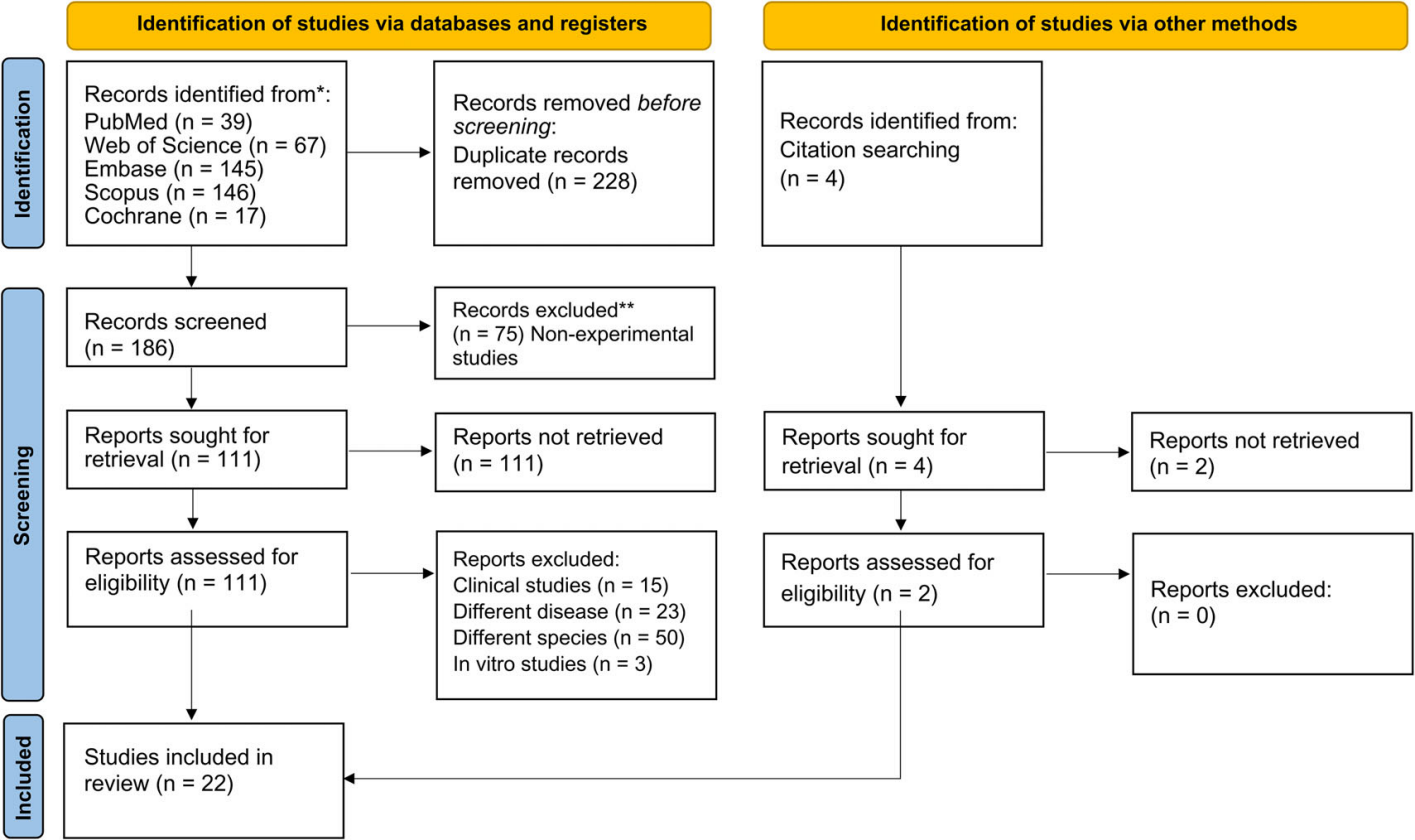
PRISMA (Preferred Reporting Items for Systematic Reviews and Meta‐Analyses) flow diagram. This flowchart illustrates the standardized stages of the search process, from identification to final inclusion of records, following the PRISMA guidelines for conducting systematic reviews and meta‐analyses. Adapted from PRISMA 2020 (http://www.prisma‐statement.org, accessed on February 26, 2025). *Consider, if feasible to do so, reporting the number of records identified from each database or register searched (rather than the total number across all databases/registers). **If automation tools were used, indicate how many records were excluded by a human and how many were excluded by automation tools. This work is licensed under CC BY 4.0. To view a copy of this license, visit https://creativecommons.org/licenses/by/4.0/.

### 3.2 Characteristics of Publications

The 22 articles have publication dates ranging from 2004 to 2025, with 4 articles published in the 2000s, 12 in the 2010s, and 6 articles in the 2020s. Brazil was the predominant country in publications with 68.18% (*n* = 15) of studies preclinical in vivo on Passiflora in DM, India next with 13.63% (*n* = 3), and then comes China, Philippines, Indonesia, and Malaysia with 4.54% (*n* = 1) each. Approval for animal use in experimental procedures by the Animal Use and Care Committee was mentioned in 81.8% of the studies, while statistical analysis was specified in 86.36% of the studies.

### 3.3 Characteristics of the Animal Model

The most of the studies (86.36%, *n* = 19) used rats as experimental models, predominantly of the Wistar lineage (17 studies). Mice were used in 13.63% (*n* = 3) of the studies, with two employing the genetically modified db/db strain (Figure 2a). Regarding sex distribution, 81.81% (*n* = 18) of the studies used male animals, 13.63% (*n* = 3) included both sexes, and 4.54% (*n* = 1) did not report the sex of the animals (Figure 2b). In terms of age, 68.18% (*n* = 15) of the studies used adult animals, while 13.63% (*n* = 3) included young rats (up to 8 weeks old, as defined by Jackson et al., 2017 [13]). Four studies (18.18%) did not report the animals' age. Among the 19 studies that used rats, body weight ranged from 140 g to 250 g in 11 studies, exceeded 250 g in 7, and was not reported in 1. For mice, only one study reported a weight range of 20–30 g; the remaining two did not disclose this information. As for diabetes induction, alloxan was the most commonly used agent, applied in 68.18% (*n* = 15) of the studies, followed by streptozotocin in 22.72% (*n* = 5). Genetically diabetic db/db mice were used in 9.10% (*n* = 2) of the studies (Figure 2c).

**FIGURE 2:**
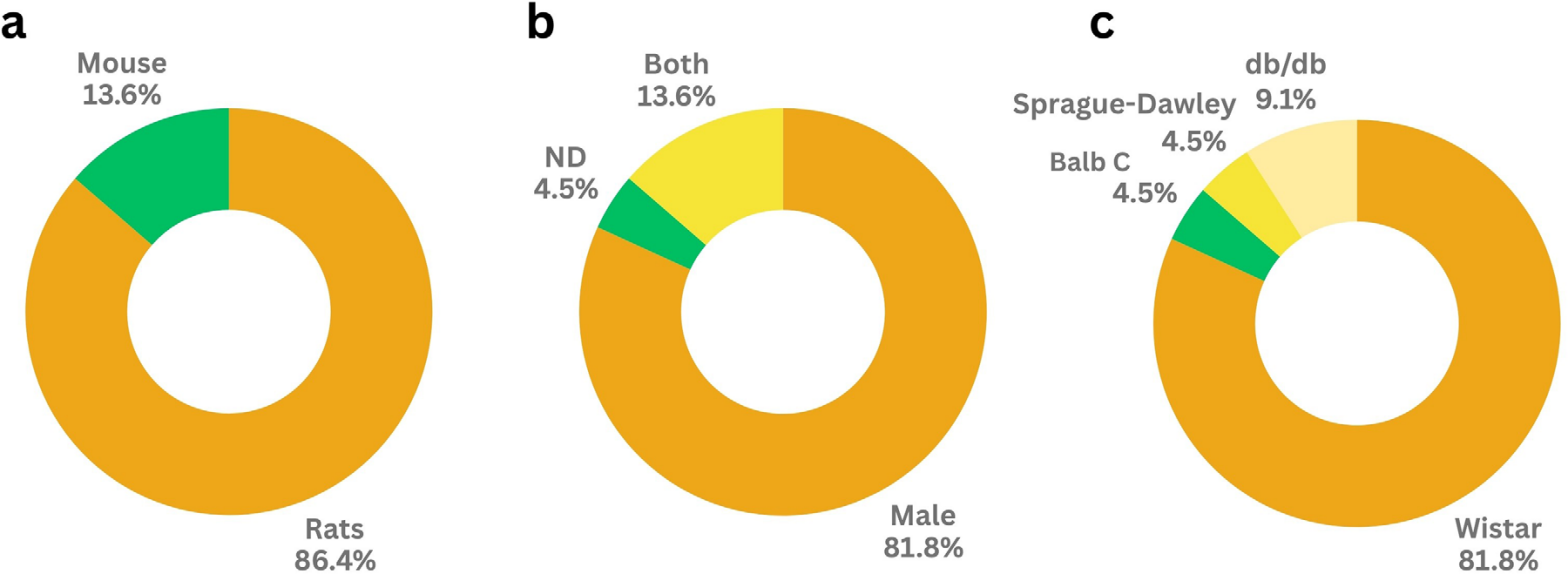
Characteristics of the animal model found in the studies. (a) Species, (b) sex, and (c) lineage.

### 3.4 Characteristics of Treatments

Among the nomenclatures used to define the passion fruits used in treatments, “*P. edulis*” was used in 45.45% (*n* = 10) of the studies, “*P. edulis* Sims” in 27.27% (*n* = 6), and “*P. edulis* fo. *flavicarpa*” in 27.27% (*n* = 6). Regarding the used parts of plants, the peel fruits were the preferred part used in 50% (*n* = 11) of the studies, followed by the leaves with 27.27% (*n* = 6), the pulp with 18.18% (*n* = 4) and 1 study (4.54%) used peels and seeds together (Figure 3A). Regarding the extraction methods used in the studies, only six studies (27.27%) did not use any, using passion fruit peel flour. Furthermore, 59.09% of studies (*n* = 13) administered extracts from different parts of the plant. In contrast, one study used an isolated compound from the peels as a treatment, two studies used fractions (a polysaccharide fraction from the peels and a butanolic fraction from the leaves).

**FIGURE 3:**
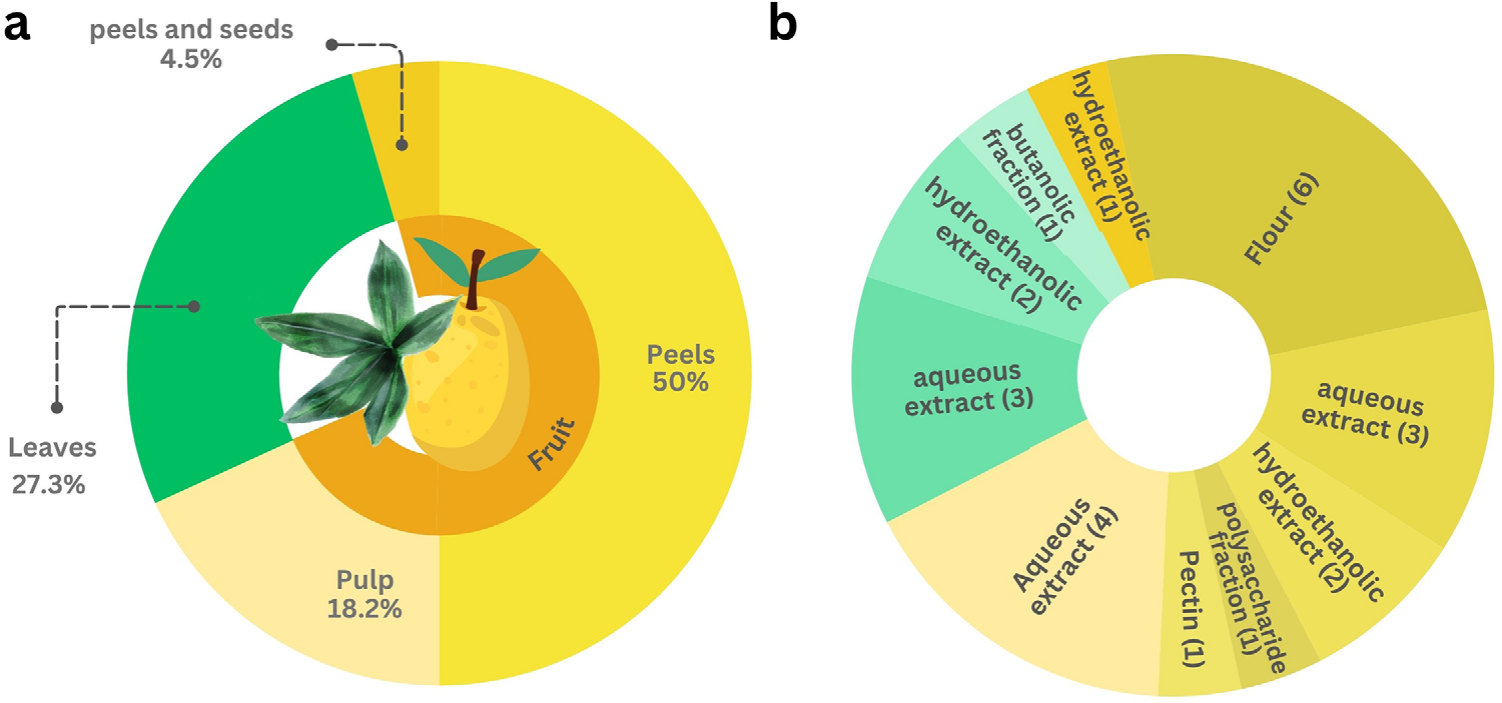
Plant parts (a) and extract preparations (b) used in studies on *Passiflora edulis*. The figures complement each other, as the colors used in the graphs symbolize the parts of *P. edulis* used and their respective extracts.

A total of 13 studies investigated extracts, but 15 extracts were produced because 2 studies [7, 14] generated 2 extracts from the peels in each study (Figure 3B). Of these 15 extracts, 33.33% (*n* = 5) used ethanol as the solvent, and all employed maceration as the extraction method. The remaining ten extracts were aqueous. Among these, four were obtained through turbo‐extraction (juice), three were obtained through decoction, one through maceration with stirring, and two studies did not report the specific extraction method used. All studies administered the extracts via the oral route.

### 3.5 Main Outcomes

Phytochemical analysis was performed in 36.36% (*n* = 8) of the included studies. Chromatographic and spectrophotometric techniques were employed to identify and quantify the chemical composition of different parts of *P. edulis*, especially the leaves and fruit peels. The main sample types analyzed included extracts and flours.

The most frequently identified class of compounds was flavonoids, cited in 31 instances across the included studies. These included *C*‐glycosylated flavones (Figure 4) such as orientin (**1**), isoorientin (**2**), vitexin (**3**), isovitexin (**4**), and vicenin‐2 (**5**). Phenolic compounds were the second most reported class (*n* = 11), primarily quantified as total phenolics using UV–vis spectrophotometry. Other compounds identified, although less frequent, included carotenoids (*n* = 3), tannins, triterpenes, steroids, and polysaccharides (*n* = 1).

**FIGURE 4:**
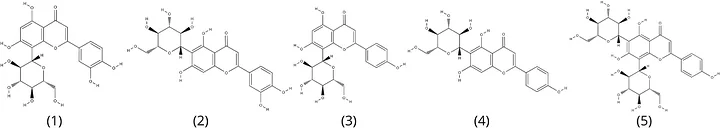
Chemical structures of the most frequently identified C‐glycosylated flavonoids in the included studies.

Table 1 summarizes the phytochemical findings of the selected studies, detailing the plant part, extraction method, identified compounds or classes, quantification results, and analytical techniques used.

**TABLE 1:** Summary of selected studies on the phytochemical composition of *Passiflora edulis*.

Author (year)	Part of the plant	Sample type	Identified compound or class	Quantification	M
[15]	Fruit peels	Flour	Total phenolics	4.20 mg g^−1^ of fresh matter	UV–Vis spectrophotometry
			Tannins	0.17 mg g^−1^ of fresh matter	
[16]	Leaves	Ethanolic extract (maceration)	Total phenolics	4.67 ± 0.115 g GAE/100 g	UV–Vis spectrophotometry
[17]	Fruit peels and seeds	Ethanolic extract (maceration)	Esteroids	ND	HPTLC
			Triterpenoids		
			Flavonoids		
[18]	Fruit peels	Flour	Carotenoids	0.321 mg/100 mg of dry weight	HPLC‐DAD
			Cryptoxanthin	2.23 mg/100 mg of dry weight	
			Lutein	57.53 mg/100 mg of dry weight	
			Total phenolics	248.9 mg GAE/100 g	UV–Vis spectrophotometry
[8]	Leaves	Ethanolic extract (maceration)	4‐*o*‐*β*‐Glucopyranosyl caffeic acid	ND	HPLC‐ESI‐IT‐MS
			Phenolic acid		
			Apigenin‐6‐*C*‐arabinoside‐8‐*C*‐glycoside		
			Isovitexin		
			Quercetin‐3‐*O*‐β‐glucopyranosyl‐7‐o‐α‐rhamnopyranoside		
			Quercetin‐8‐*O*‐β‐glucopyranosyl‐6‐*O*‐α‐rhamnopyranoside		
			6,8‐Di‐*C*‐β‐glucopyranosylapigenin (vicenin‐2)		
			Derived phenolic acid		
			Isoorientin		
			Isoorientin	3.90%	UPLC‐ESI‐MS
[7]	Fruit peels	Aqueous extract (decoction)	Total phenolics	7.71 ± 0.12 (mg of GAE/g of extract)	UV–Vis spectrophotometry
			Total flavonoids	2.65 ± 0.05 (mg of RE/g extract)	
			Orientin	ND	UHPLC/UV‐DAD
			Pectin	ND	HPSEC e NMR
		50% Ethanolic extract (maceration)	Total phenolics	10.65 ± 0.18 (mg of GAE/g of extract)	UV–Vis spectrophotometry
			Total flavonoids	3.55 ± 0.14 (mg of RE/g extract)	
			Isoorientin	ND	UHPLC/UV‐DAD
[10]	Leaves	Ethanolic extract (maceration)	Apigenin‐6,8‐di‐*C*‐glycoside	ND	HPLC‐ESI‐IT‐MS
			Apigenin‐6‐*C*‐arabinoside‐8‐*C*‐glycoside		
			Luteolin‐7‐*O*‐pyranosyl‐3‐*O*‐glycoside		
			Isoorientin		
			Isovitexin		
			Isoorientin (repeated)	6.10%	UPLC‐ESI‐MS
			Total flavonoids	11.01%	
[14]	Fruit peels	Aqueous extract (decoction)	Vicenin‐2	81.1 µg/g	LC‐QqQ‐MS/MS
			Orientin	205.7 µg/g	
			Isoorientin	204.4 µg/g	
			Vitexin	34.3 µg/g	
			Isovitexin	33.9 µg/g	
		Aqueous extract (decoction)	vicenin‐2	97.8 µg/g	
			Orientin	270.4 µg/g	
			Isoorientin	304.3 µg/g	
			Vitexin	33.5 µg/g	
			Isovitexin	34.7 µg/g	

**Abbreviations**: GAE, gallic acid equivalents; HPLC‐DAD, high‐performance liquid chromatography with diode array detector; HPLC‐ESI‐IT‐MS, high‐performance liquid chromatography with electrospray ionization and ion trap mass spectrometry; HPSEC, high‐performance size‐exclusion chromatography; HPTLC, high‐performance thin‐layer chromatography; LC‐QqQ‐MS/MS, liquid chromatography coupled to triple quadrupole tandem mass spectrometry; ND, not determined; RE, rutin equivalents; RMN, nuclear magnetic resonance; UHPLC/UV‐DAD, ultra‐high‐performance liquid chromatography with ultraviolet diode array detector; UPLC‐ESI‐MS, ultra‐performance liquid chromatography with electrospray ionization and mass spectrometry; UV–vis, ultraviolet–visible spectrophotometry.

Among the doses reported, studies employing the aqueous leaf extract most frequently used 200 mg/kg (*n* = 3), while 100, 300, and 400 mg/kg were each reported once. For the hydroethanolic leaf extract, 200 mg/kg was the most common dose, described in two studies. Regarding the fruit peel, it was necessary to distinguish between studies that produced extracts and those that used the flour. Among the peel extracts, the aqueous extract was administered three times at 400 mg/kg and once at 600 mg/kg, whereas the hydroethanolic extract was used once at 400 mg/kg and once at 600 mg/kg. For studies that administered peel flour directly, three delivery methods were identified: gavage, diet formulation, and dissolution in drinking water. In gavage, doses of 20, 40, 160, 215, 430, and 1500 mg/kg were each tested once. In diet formulations, the proportion of flour in the feed varied: 10–15% was used three times, while 0–9% and 30% were reported once each. The study that dissolved the flour in drinking water did not specify the resulting concentration. Additionally, from peel‐derived fractions, one study evaluated isolated pectin at doses of 2, 10, and 25 mg/kg, while another assessed the polysaccharide fraction at 50, 100, and 200 mg/kg.

The 22 studies included in this review, markers of glycemic control, such as blood glucose, glycated hemoglobin (HbA1c), and protein glycation, were the most frequently evaluated outcomes. A total of 81.81% (*n* = 18) of the studies reported at least one of these parameters, with two demonstrating a reduction in HbA1c and three reporting decreased protein glycation. The passion fruit peel was the most commonly used plant part for this purpose, cited in 10 studies, followed by the leaves (5), pulp (2), and a combination of peel and seeds (1).

Additionally, increased insulin levels and improved insulin sensitivity were observed in two studies, while two others reported elevated glycogen levels. It is important to note that most studies assessed multiple biological activities of *P. edulis*, not exclusively glycemic control.

Improvement in lipid profile was the second most frequently reported outcome, observed in 40.9% (*n* = 9) of the studies. This was evidenced by reductions in total cholesterol and triglyceride levels, increased high‐density lipoprotein (HDL), and decreased leptin in one study. Again, the peels were the most frequently associated with this effect (four studies), followed by leaves (three) and pulp (two).

Antioxidant activity ranked third, reported in 36.36% (*n* = 8) of the studies. Most of these involved leaf extracts (four studies), with peels (two), pulp (one), and a combination of peel and seeds (one) also represented. Antioxidant effects were mainly demonstrated through inhibition of reactive oxygen species (ROS), reduction of oxidative stress marker malondialdehyde (MDA), and enhancement of enzymatic (e.g., superoxide dismutase and catalase) and non‐enzymatic (e.g., glutathione) antioxidant defenses.

Other less frequently reported outcomes included improved kidney function (four studies), decreased liver injury markers (three studies), and histological evidence of tissue restoration (three studies), corresponding to 13.63% of the total studies for each.

These findings are summarized in Figure 5, which illustrates the primary biological activities associated with different parts of *P. edulis* investigated in the reviewed studies.

**FIGURE 5:**
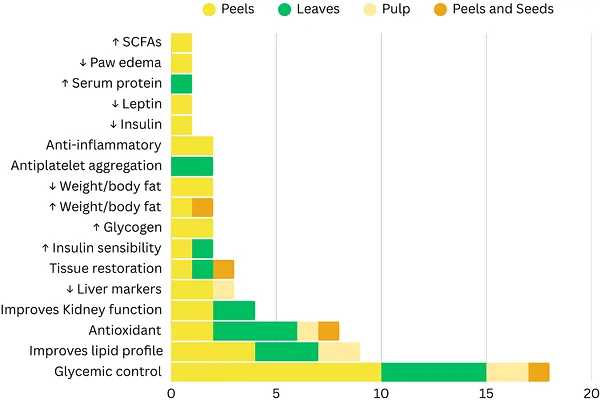
Primary outcomes produced by *P. edulis* are found in the studies.

Based on the integrated analysis of the included studies, we propose a hypothetical and integrative mechanistic model for *P. edulis*, combining experimentally observed outcomes with pathways inferred from the broader scientific literature (Figure 6). In the intestinal lumen, pectin slows the absorption of glucose and lipids by forming viscous gels and inhibiting key digestive enzymes such as α‐amylase and α‐glucosidase, as well as by modulating glucose transporters (SGLT‐1 and GLUT2). Concurrently, the fermentation of pectin by gut microbiota generates short‐chain fatty acids (SCFAs), which stimulate the secretion of incretin hormones (GLP‐1 and PYY), promoting satiety, enhancing insulin secretion, and further attenuating nutrient absorption.

**FIGURE 6:**
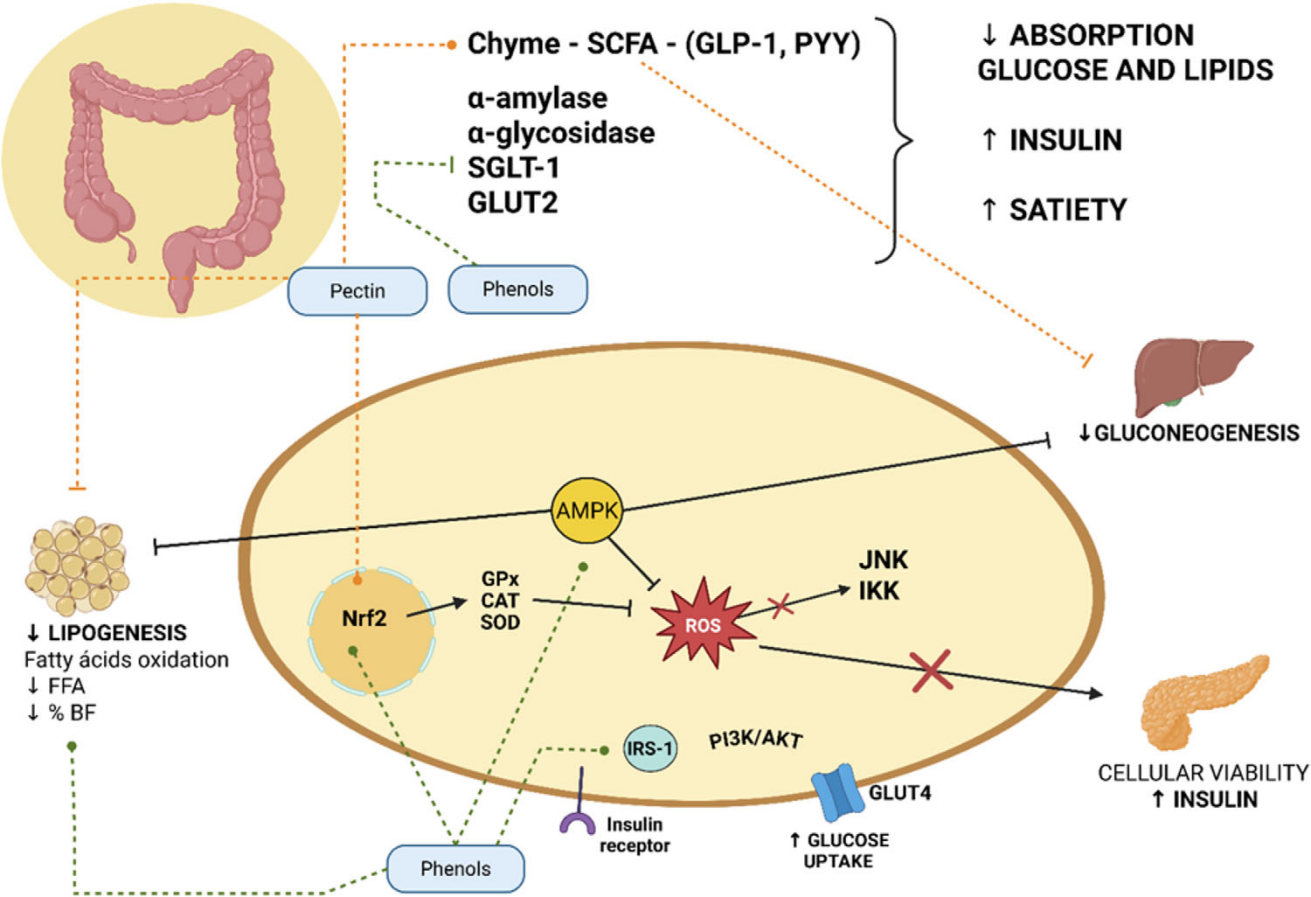
Hypothetical and integrative schematic representation of the proposed mechanisms of *Passiflora edulis* in diabetic models. SGLT‐1 (sodium‐glucose cotransporter 1); GLUT2 (glucose transporter 2); GLUT4 (glucose transporter 4); GLP‐1 (glucagon‐like peptide 1); PYY (peptide yy); ROS (reactive oxygen species); GPx (glutathione peroxidase); CAT (catalase); FFA (free fatty acids); SOD (superoxide dismutase); IRS‐1 (insulin receptor substrate 1); BF (body fat); Nrf2 (nuclear factor erythroid 2‐related factor 2).

At the cellular level, phenolic compounds activate AMP‐activated protein kinase (AMPK), leading to increased glucose uptake (via GLUT4 translocation), suppression of hepatic gluconeogenesis, and enhanced lipid oxidation. These effects contribute to reduced lipogenesis, lower body fat percentage, and improved insulin sensitivity. Additionally, flavonoids stimulate the Nrf2 signaling pathway, enhancing the expression of antioxidant enzymes such as GPx, CAT, and SOD, thereby mitigating oxidative stress (ROS).

Moreover, phenolics inhibit pro‐inflammatory pathways (JNK and IKK), which are typically associated with insulin resistance through the aberrant phosphorylation of IRS‐1. By preserving the PI3K/AKT signaling cascade, these compounds help maintain insulin signaling and glucose homeostasis.

### 3.6 Reporting Bias in Studies

The results of the bias analysis using SYRCLE's tool are represented in Figures 7 and 8. Of the 13 items evaluated, only 4 had the presence of “unclear”; the others were evaluated with “yes” (low risk) or “no” (high risk). The items most frequently rated as “yes” (thus indicating low risk of bias), were extract preparation and other biases (90.9%; *n* = 20), incomplete outcome data and statistical methods (86.4%; *n* = 19), ethical approval (81.8%; *n* = 18), selective reporting and baseline characteristics (77.3%; *n* = 17). Items categorized as having an unclear risk of bias included: random sequence generation and statistical methods (9%; *n* = 2), as well as incomplete outcome data and other bias (4.5%; *n* = 1). Conversely, the domains with the most critical evaluations, rated predominantly as “no” (indicating high risk of bias), were allocation concealment and blinding of participants and personnel (100%; *n* = 22), blinding of outcome assessment and pharmacological evaluation of the isolated compound (95.4%; *n* = 21), random sequence generation and phytochemical characterization (59%; *n* = 13). Overall, the methodological quality of the included studies was considered acceptable, with a predominance of low risk of bias in key aspects such as extract preparation, animal baseline characteristics, and the completeness of outcome data. However, some gaps remain, particularly in studies involving natural products. Critical limitations include the lack of detailed information on extract preparation, the identification and specification of plant species and parts used, and the absence of phytochemical characterization of the plant material or extract.

**FIGURE 7:**
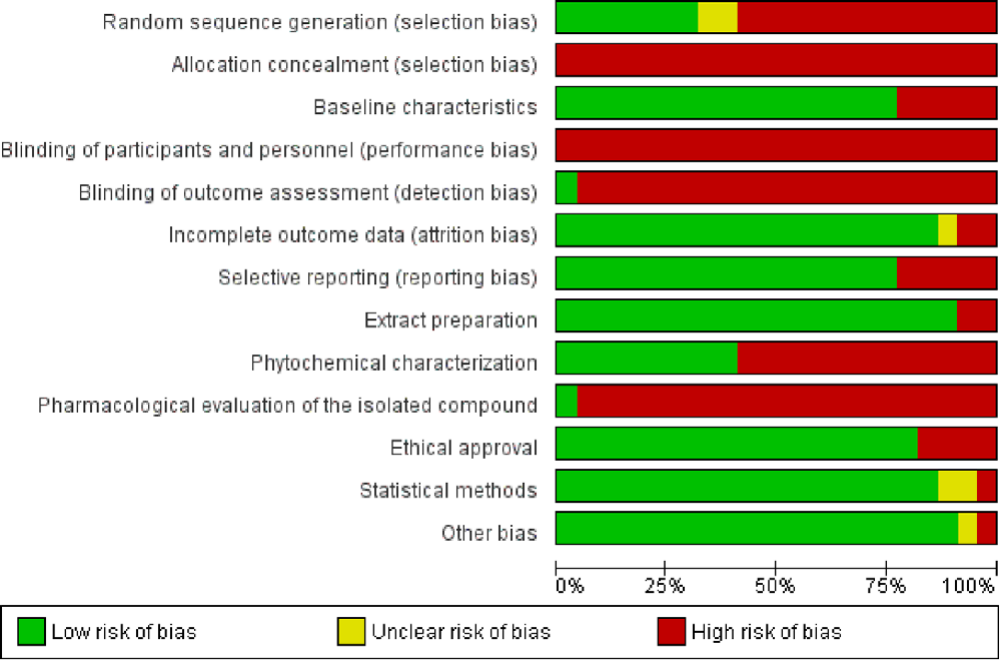
Results for risk of bias and methodological quality indicators for all in vivo studies included in this systematic review that evaluated the effects of *Passiflora edulis* on diabetes mellitus. Items in the SYRCLE Risk of Bias assessment were scored as “yes” indicating low risk of bias (green), “no” indicating high risk of bias (red), or “unclear” indicating that the item was not reported.

**FIGURE 8:**
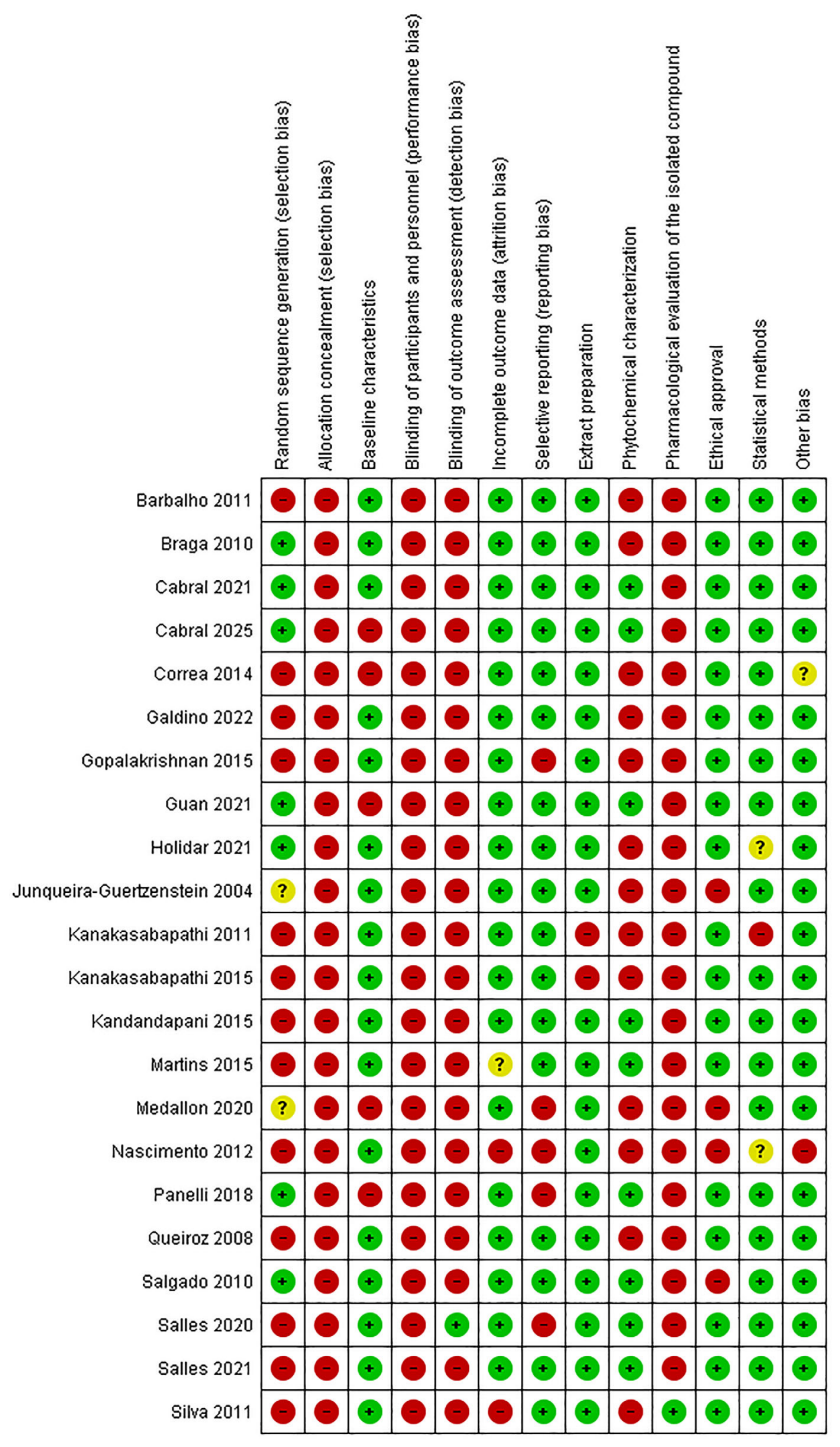
Risk of Bias summary: Authors’ judgments of the risk of bias items for each included study. Green: low risk of bias; yellow: unclear risk of bias; red: high risk of bias.

## 4 Discussion

### 4.1 Relevance of *Passiflora edulis* and Characteristics of Included Studies

*Passiflora edulis* species holds significant commercial and therapeutic relevance. Its fruits, particularly cultivated in South America, are known for their nutritional value, while their leaves have been traditionally used in Latin American medicine, mainly for their sedative properties. The fruit itself has gained attention in the treatment of diabetes mellitus (DM), which has led to investigations into its nutraceutical and pharmacological properties. Studies have reported protective effects against reactive oxygen species (ROS) and protein glycation, as well as antihyperglycemic and anti‐inflammatory activity, due to its high content of flavonoids and pectins [6, 19].

This systematic review evaluated the effects of *P. edulis* in preclinical in vivo models of DM and included 22 heterogeneous studies. Overall, the evidence suggests that the species exhibits some efficacy either as a treatment or as an adjuvant in glycemic control.

Among the included studies, six employed *P. edulis* fo *flavicarpa* (yellow passion fruit) as the intervention, while another six specified *P. edulis* fo *edulis* (purple passion fruit). However, nearly half of the studies (*n* = 10) did not report the taxonomic form of the species investigated. This omission is a relevant limitation, since previous evidence has demonstrated that the forms of *P. edulis* exhibit significant genetic and phytochemical differences [5, 19], which may underlie distinct pharmacological properties. Therefore, intra‐specific taxonomic characterization is essential in studies involving this species.

It is also necessary to emphasize that it is important to consider the plant by distinguishing its different parts. Among the 22 studies analyzed, 16 evaluated fruit‐based preparations, while only 6 investigated the leaves. Notably, the fruit peel was the most frequently used plant part, indicating its potential as a therapeutic target. The pulp was studied in four articles, and one study included both peel and seeds.

This emphasis on the peel also brings attention to its relevance in sustainability. It is known that only 35%–40% of passion fruit mass is used for juice extraction, while 60%–65% consists of peels and seeds, often discarded. Globally, fruit processing generates approximately 0.5 billion tons of agro‐industrial waste each year [20]. There is growing interest in the sustainable reuse of such waste products in the development of animal feed, edible oils, pharmaceuticals, nutraceuticals, and cosmetics [21, 22]. In this context, *P. edulis* peel emerges as a bio‐residue with high potential for valorization.

Regarding geographic distribution, Brazil accounted for 15 of the included studies. This is consistent with Brazil's standing as the world's leading producer of passion fruit, responsible for over 65% of global production [23]. Brazil is known for its traditional use of medicinal plants to treat various diseases, including DM. *Passiflora edulis* is included in the Brazilian list of medicinal plants of interest to the Unified Health System (RENISUS), encouraging scientific research on its properties. The remaining seven studies were distributed among India, China, the Philippines, Indonesia, and Malaysia, regions where passion fruit is naturally found in tropical and subtropical climates [24].

All studies included in the review used rodent animals (rats and mice) as in vivo models. This is due to the several advantages of using these animals as experimental models, such as the fact that they are mammals that share physiological/metabolic similarities with humans, high reproductive rate, low maintenance cost, easy handling, as well as the possibility of being able to analyze tissue and biochemical measurements in all stages of diabetes mellitus [25]. All studies administered the extracts via the oral route, which aligns with its relevance to glycemic control and the role of gut hormones such as GLP‐1 in modulating glucose tolerance [26].

Most of the rodents used in the studies were male, probably due to the assumption that the females' estrous cycle makes them more variable in their biological parameters. Although there is important research that highlights the problem of underutilization of female animals in science and that there is no differentiation in relation to males in several biological characteristics [27, 28], it is already known that there are sexual differences with regard to male and female responses to DM, being therefore, it would be relevant for authors who used both sexes to discuss likely differences between the groups or to consider them in the results [29, 30].

Regarding the diabetogenic drug used in the studies, alloxan was a most used drug; certainly, due to its low cost and because it is cheaper and easy to acquire. However, some papers show that this diabetes induction model has limitations regarding its instability and effectiveness, and that streptozotocin has several advantages in relation to diabetogenicity, being, therefore, a better drug choice in studies with diabetes in animal models [31]. Although there are other genetically modified animal models for studies on diabetes [32], two of our studies opted for db/db mice, which are animals that contain a mutation in the leptin receptor gene, which gives them morbid obesity, chronic hyperglycemia, pancreatic beta cell atrophy, and hypoinsulinemia, being a good model of DM2 [33].

In summary, the current body of evidence supports *P. edulis* as a promising species in the context of diabetes research, particularly with respect to its fruit peel. The following section explores in greater detail the specific pharmacological effectiveness of different plant parts and their preparation methods.

### 4.2 Plant Parts and Extracts: Effectiveness in Glycemic Control

Based on the main results presented (Figure 5), it is possible to state that the fruit of *P. edulis*, especially the peel, represents the most effective part of the plant in terms of glycemic control. The peels are investigated in this context and are commonly used in the form of flour in studies that evaluate their effects as a nutritional supplement. It is believed that this effect is predominantly attributed to the presence of soluble fibers, especially pectin, mostly found in this fraction of the fruit.

Recent evidence indicates that the aqueous extract of the peels of *P. edulis* may have superior hypoglycemic activity to that of the flour, possibly due to a higher yield in pectin extraction. In a study conducted by Silva (2011) [34], the administration of isolated pectin extracted from the peels demonstrated significant efficacy, with reductions of up to 65% in glycemic levels at doses of 2, 10, and 25 mg/kg in Wistar rats, a performance superior to that observed in other studies covered in this review. Cabral (2021) [7] evaluated and compared the effects of the hydroethanolic extract by maceration and the aqueous extract obtained by decoction of the peels, demonstrating that the latter presented a concentration of 34.3% pectin, substantially higher than the 19.8% content described by Yapo and Koffi (2006) for the peel flour form [35]. In addition, the aqueous extract produced a more pronounced hypoglycemic response compared to the ethanolic extract. These data reinforce the hypothesis that the aqueous extraction method optimizes the availability and functional use of pectin, possibly amplifying its beneficial effects in the context of glycemic control.

These findings reinforce the hypothesis that not only is the peel the most pharmacologically active part of the fruit, but also that the extraction method plays an important role in maximizing its bioactive potential. The decoction process, in particular, appears to enhance the extraction of active components, leading to stronger therapeutic effects.

### 4.3 Mechanisms of Action of Bioactive Compounds Identified in *Passiflora edulis*

Although pectin appears to play a relevant role in glycemic modulation, it is unlikely that it is solely responsible for this effect. Studies conducted with the pulp [9, 36] and the leaves of the plant [8, 10, 16, 37] also demonstrated significant hypoglycemic activity, suggesting the action of other bioactive compounds. Corroborating this hypothesis, Cabral (2021) demonstrated that the hydroethanolic extract of the peel, even without the detectable presence of pectin, was effective in reducing glycemia in experimental models of type 1 diabetes [7]. These results indicate that secondary metabolites, such as phenolic compounds and flavonoids, also play an important role in the antidiabetic properties of the plant. In addition to glycemia modulation, both lipid control and antioxidant activity (Figure 5) are processes strongly associated with the pathophysiology of type 2 diabetes mellitus [38]. In this context, in addition to pectin, the phenolic and flavonoid compounds identified in the studies (Figure 4) present in *P. edulis* play a central role in promoting these effects.

Pectin, a soluble fiber of prebiotic nature present in the skin of the passion fruit, is widely recognized for its ability to form a viscous gel in the intestinal lumen, which significantly slows down the digestion and absorption of carbohydrates and lipids [39]. However, in addition to this physicochemical effect, pectin has multiple metabolic actions with a direct impact on the control of diabetes mellitus [40].

As seen by Guan (2021) [41], during its fermentation in the colon by beneficial bacteria of the intestinal microbiota, pectin gives rise to the production of short‐chain fatty acids (SCFAs), such as butyrate, propionate, and acetate [42]. These metabolites act as important metabolic signaling, promoting the inhibition of hepatic gluconeogenesis and increased insulin sensitivity [43]. In addition, pectin stimulates the secretion of the intestinal hormones GLP‐1 (glucagon‐like peptide‐1) and PYY (peptide YY), which are involved in increasing satiety, regulating insulin secretion, and reducing caloric intake, contributing additionally to metabolic control [44].

Regarding lipid metabolism, pectin exerts beneficial effects by reducing intestinal lipid absorption and modulating the expression of hepatic enzymes involved in lipid synthesis, contributing to improving the plasma lipid profile and reducing cardiovascular risk [45].

In addition, pectin has indirect antioxidant activity, particularly through activation of the nuclear factor erythroid 2‐related factor 2 (Nrf2) pathway, which induces the expression of genes encoding antioxidant enzymes such as superoxide dismutase (SOD), glutathione peroxidase (GPx), and catalase [46]. This activation contributes to the neutralization of reactive oxygen species (ROS) and the protection of tissues sensitive to oxidative damage, such as pancreatic β cells, kidneys, and the peripheral nervous system.

Flavonoids such as rutin, apigenin and quercetin, as well as pectin, exert inhibitory effects on enzymes such as α‐amylase and α‐glucosidase, reducing glucose absorption in the intestine [40, 47]. Furthermore, flavonoids such as naringenin and epicatechin can compete with glucose for intestinal transporters, such as sodium‐glucose co‐transporter 1 (SGLT‐1) and GLUT2 [48, 49], inhibiting glucose absorption and, consequently, postprandial glycemic peaks.

At the cellular level, bioactive compounds from *P. edulis* have been reported to influence pathways associated with insulin resistance, inhibition of gluconeogenesis, and increased insulin secretion. However, it is important to note that most of these mechanisms were not directly investigated in the included studies and are inferred from supporting literature. Compounds such as quercetin, luteolin and kaempferol promote glucose uptake by activating insulin receptors and insulin receptor substrate 1 (IRS‐1) triggering the PI3K/AKT pathway causing the translocation of the GLUT4 receptor to the membrane [50]. Sun and collaborators (2024) demonstrated that pectin also has the ability to modulate this pathway.

An additional mechanism of some phenolic compounds, such as vitexin and orientin, resides in the ability to activate AMP‐activated protein kinase (AMPK), a key enzyme in energy homeostasis [51, 52]. AMPK activation is associated with increased glucose uptake in liver and muscle tissues, partly overlapping with insulin signaling pathways such as PI3K/AKT. In addition, it reduces oxidative stress, inhibits hepatic gluconeogenesis, and performs lipid control through fatty acid oxidation and lipogenesis suppression, contributing to improving the metabolic profile. As reported by Cabral et al. (2021) [7], the administration of *P. edulis* extract in association with insulin showed effects comparable to insulin combined with metformin in glycemic control. However, this observation should be interpreted with caution, as it is based on a single experimental model and lacks standardized comparative design across studies. The involvement of pathways such as AMPK activation remains a plausible mechanism based on the known biological activity of phenolic compounds [53], although this was not directly demonstrated in most of the included studies.

In addition to metabolic regulation, oxidative stress plays a central role in the pathophysiology of DM, contributing to insulin resistance (IR), pancreatic β‐cell dysfunction, and the development of chronic diabetic complications. Phenols and flavonoids are considered potent antioxidants due to their ability to neutralize ROS, which activate inflammatory pathways such as JNK and IKK‐β. These kinases promote serine phosphorylation of IRS‐1, impairing insulin signaling and contributing to insulin resistance [54]. ROS also enhance inflammatory responses by activating transcription factors such as NF‐κB, leading to the production of pro‐inflammatory cytokines. These cytokines further activate serine kinases, including JNK and IKK‐β, exacerbating the impairment of IRS‐1 signaling and worsening insulin resistance [55]. In addition, flavonoids have been shown to induce antioxidant enzymes and activate the Nrf2 pathway, enhancing cellular defense against oxidative stress [56]. This antioxidant activity also protects pancreatic β cells from dysfunction and apoptosis, as these cells are particularly susceptible to oxidative damage.

Phenols and flavonoids can modulate lipid metabolism and reduce adipose tissue, which when in excess is strongly related to the development and progression of DM. Compounds such as isoorientin and quercetin can activate AMPK, increasing the oxidation of fatty acids in liver and muscle tissue [57]. Flavonoids also regulate the gene expression of transcription factors related to lipid metabolism, such as PPARα and SREBP‐1c, causing both fatty acid oxidation and inhibition of lipogenesis [58]. In addition, they are able to inhibit lipid synthesis enzymes, reduce plasma levels of low‐density lipoprotein (LDL) [59] and reduce its absorption in the intestine. These activities tend to reduce the concentration of free fatty acids, body fat percentage, oxidative stress and release of pro‐inflammatory cytokines, improving insulin resistance, in addition to reducing the cardiovascular risk associated with diabetes. Altogether, these findings demonstrate that the therapeutic potential of *P. edulis* arises from the synergistic interaction of multiple bioactive compounds acting across complementary metabolic pathways.

Importantly, the interpretation of the reported antihyperglycemic and metabolic effects should be considered in light of the methodological limitations identified by the SYRCLE risk of bias assessment. The consistent absence of allocation concealment and blinding across studies introduces a high risk of performance and detection bias, which may lead to an overestimation of treatment effects. Additionally, the lack of standardized phytochemical characterization further limits reproducibility and mechanistic interpretation.

### 4.4 Limitations and Gaps

Several important limitations were identified across the included studies, which should be considered when interpreting the findings of this review. A major concern is the lack of standardization in experimental design, particularly regarding extraction methods, plant material, and dosing regimens. The wide variability in administered doses and treatment protocols precludes the establishment of consistent dose–response relationships and limits the comparability between studies.

Another critical limitation is the insufficient phytochemical characterization of the extracts used. As bioactive compounds such as pectin, phenolics, and flavonoids are likely responsible for the observed biological effects, the absence of detailed compositional analysis significantly compromises reproducibility and mechanistic interpretation.

Furthermore, the use of animals of both sexes in some studies represents a significant source of bias, since sex‐related differences in metabolic responses are well documented but remain underexplored in this context. The lack of standardized comparators, including the inconsistent use of reference antidiabetic drugs, further limits the interpretation of the relative efficacy of *P. edulis*.

Variability in diabetes induction models, including the use of alloxan, streptozotocin, and genetically modified models, also contributes to heterogeneity in outcomes and may influence the observed effects, reducing the overall translational relevance to human type 2 diabetes.

Furthermore, toxicity and safety parameters were not systematically assessed across studies, representing a significant gap in the evaluation of the therapeutic potential of *P. edulis*. Finally, methodological limitations identified through the SYRCLE risk of bias assessment, particularly the lack of allocation concealment and blinding, may have contributed to an overestimation of treatment effects. Collectively, these limitations highlight the need for more standardized, well‐controlled, and mechanistically oriented studies to improve the reliability and translational applicability of the current evidence.

## 5 Conclusion

In summary, the in vivo preclinical evidence analyzed suggests that *P. edulis* may exert beneficial effects in the context of diabetes mellitus, with emphasis on the use of its peel, particularly in the form of aqueous extract. Most studies indicate hypoglycemic activity, along with favorable effects on lipid metabolism and oxidative stress. Considering the phytocomplex nature of the extracts, these effects may be associated with the presence of pectin and secondary metabolites such as phenolic compounds and flavonoids. The proposed mechanisms of action appear to be multifactorial, including modulation of intestinal glucose absorption and pathways such as PI3K/AKT and AMPK, although these mechanisms were not consistently demonstrated in the included studies. However, the significant variability in dosing, extraction methods, and experimental design, along with limited phytochemical characterization, represent important methodological limitations that restrict the robustness and reproducibility of the findings. Overall, this review highlights the potential of passion fruit peels as a bioactive resource, while emphasizing the need for more standardized and mechanistically oriented studies to support its translational application in diabetes management. These advances will be essential to clarify the therapeutic relevance of *P. edulis* and guide its future clinical investigation.

## Author Contributions

**Luiz Ricardo Teixeira de Farias**: writing – original draft, data curation, validation. **Mariáurea Matias Sarandy**: data curation, methodology. **Reggiani Vilela Gonçalves**: data curation, validation, supervision. **Emanuella de Aragão Tavares**: writing – review and editing, data curation, supervision. **Silvana Maria Zucolotto**: writing – review and editing, conceptualization, supervision.

## Funding

Funding for this research was provided by the National Council for Scientific and Technological Development (CNPq, Brazil) through a research productivity fellowship to Silvana Maria Zucolotto (grant no. 313727/2020–1) and by the Coordination for the Improvement of Higher Education Personnel (CAPES, Brazil) through a postdoctoral fellowship to Emanuella de Aragão Tavares (grant no. 88887.082421/2024‐00).

## Conflicts of Interest

The authors declare no conflicts of interest.

## Supporting information




**Supporting File 1**: cbdv71700‐sup‐0001‐SuppMat.docx

## Data Availability

The data that supports the findings of this study are available in the Supporting Information of this article.
