# Supplementary material for: A Systematic Review of Antidiabetic Mechanisms of Passiflora edulis: Beyond Glycemic Control
Source: Chem Biodivers. 2026 Oct 20;23(10):e71700. doi: 10.1002/cbdv.71700 (PMC13682687; doi:10.1002/cbdv.71700)
Supplement: Supplementary file 1 [file CBDV-23-e71700-s001.docx]

**Suplemmentary Material**

**Table S1**: Table of descriptors used and items found in the Embase, Web of Science and PubMed-Medline databases.

| **Data Base** | **Descriptors** | **Items Found** | **Time** | **Date** |
| --- | --- | --- | --- | --- |
| **Embase** | 'passiflora'/exp OR 'passiflora' OR 'passiflora edulis'/exp OR 'passiflora edulis' OR 'passion fruit'/exp OR 'passion fruit' OR 'maracuja' | 2.641 | 16:58 | 07/04/2026 |
|  | 'diabetes'/exp OR 'diabetes' OR 'diabetes mellitus'/exp OR 'diabetes mellitus' | 1.936.972 | 16:58 | 07/04/2026 |
|  | **TOTAL #1 AND #2** | **145** | **16:59** | **07/04/2026** |
| **Web of science** | TS=Passiflora OR TS=Passiflora edulis OR TS=passion fruit OR TS=maracuja | 5.374 | 17:03 | 07/04/2026 |
|  | TS=diabetes mellitus OR TS=diabetes | 945.022 | 17:04 | 07/04/2026 |
|  | **TOTAL #1 AND #2** | **67** | **17:06** | **07/04/2026** |
| **Pubmed** | ("passiflora"[MeSH Terms] OR passiflora edulis[TIAB] OR passion fruit[TIAB] OR maracuja [TIAB]) | 1.293 | 17:10 | 07/04/2026 |
|  | "diabetes mellitus"[MeSH Terms] OR "diabetes"[Title/Abstract] OR "diabetes mellitus"[Title/Abstract] | 925.803 | 17:10 | 07/04/2026 |
|  | **TOTAL #1 AND #2** | **39** | **17:11** | **07/04/2026** |
| **Cochrane** | (passiflora) OR (passiflora) OR (passiflora edulis) OR (passiflora edulis) OR (passion fruit) OR (passion fruit) OR (maracuja) | 144 | 17:13 | 07/04/2026 |
|  | (diabetes) OR (diabetes) OR (diabetes mellitus) OR (diabetes mellitus) | 125.615 | 17:15 | 07/04/2026 |
|  | **TOTAL #1 AND #2** | **17** | **17:16** | **07/04/2026** |
| **SCOPUS** | TITLE-ABS-KEY ( passiflora ) OR TITLE-ABS-KEY ( passiflora edulis ) OR TITLE-ABS-KEY ( passion fruit ) OR TITLE-ABS-KEY ( maracuja ) | 6.866 | 17:20 | 07/04/2026 |
|  | TITLE-ABS-KEY ( diabetes ) OR TITLE-ABS-KEY ( diabetes mellitus ) | 1.381.412 | 17:21 | 07/04/2026 |
|  | **TOTAL #1 AND #2** | **146** | **17:22** | **07/04/2026** |

Source: Research data (2026)

**Table S2:** Characteristics of experimental studies that investigated the effects of Passiflora edulis in animal models of diabetes mellitus.

| Reference (Author, Year) | Plant Part Used | Extract Type | Experimental Design | Summary of Main Effects | Proposed Mechanisms |
| --- | --- | --- | --- | --- | --- |
| Junqueira-Guertzenstein, 2004 | Peels | Flour (bleached and unbleached) | 6 groups (normal and diabetic; with and without flour treatment). Blood glucose measurement and weight. Treatment: 28 days. | Reduction of blood glucose in normal and diabetic rats, especially with unbleached flour. Weight gain in treated diabetics. | Pectin delays glucose absorption. More evident effect with unbleached flour. |
| Queiroz et al., 2008 | Peels | Commercial flour | 5 groups: control, DM, DM + 50%, 100%, 150% RDI fiber. Glucose, lipid and glycogen levels, weight and organ harvesting Treatment: 30 days. | Reduced glucose, increased hepatic and cardiac glycogen. No lipid profile changes. | Action of soluble fibers on glucose absorption and increased insulin secretion, in addition to reducing glycogenolysis and gluconeogenesis. |
| Braga et al., 2010 | Peels | Powder (flour) | Groups with doses of 20, 40, 160 mg/kg. Glycemia measured at 0, 1, 2, 4, 6h. | Dose-dependent antihyperglycemic effect; best at 160 mg/kg; reversible after 6h. | Formation of a gel by fibers in the intestine; SCFAs (butyrate and propionate) reduce insulin resistance and stimulate glycogenesis. |
| Salgado et al., 2010 | Peels | Powder (flour) | 5 groups (n = 8): DM with different % of flour (5, 10, 15). Consumption, weight, blood glucose and glycogen. Treatment: 30 days. | Significant glycemia reduction with 5% and 10%; increased liver glycogen; fiber, tannin, and mineral content confirmed. | Soluble fibers and phenolic compounds reduce glucose absorption, interaction with intestinal enzymes and increase insulin sensitivity. |
| Barbalho et al., 2011 | Pulp | Pulp juice | Offspring of healthy or diabetic mothers treated with pulp juice or water. glycemic and lipid profile analysis. Treatment: 30 days. | Reduced glycemia, total cholesterol, LDL, TG and increased HDL in offspring of both healthy and diabetic mothers. | Bioactive pulp compounds act to prevent metabolic disorders in offspring of diabetic mothers. |
| Silva et al., 2011 | Peels | Isolated pectin | Diabetic rats treated with 0.5–100 mg/kg. Glycemic, lipid, and inflammatory assessments. Treatment: 5 days | Dose-dependent reduction of glycemia and triglycerides; anti-inflammatory effect (reduced paw edema, MPO, TNF-α); no added effect above 25 mg/kg. | Gastric gel formation delays emptying; reduced carb absorption; anti-inflammatory and antioxidant effects of pectin. |
| Kanakasabapathi et al., 2011 | Leaves | Aqueous | 2 tests (normal and diabetic), multiple extract doses, glibenclamide control. GTT test. | Hypoglycemic effect, best at 200 mg/kg (47% reduction). Comparable to glibenclamide. | Flavonoids and phenols as hypoglycemic agents. Effect similar to glibenclamide. |
| Nascimento & Sabaa-Srur, 2012 | Pulp | Pulp juice | Normal and diabetic rats treated with juice sweetened with sucrose or cyclamate/saccharin. Blood glucose assessment. Treatment: 21 days. | Artificial sweeteners did not worsen hyperglycemia in diabetics. Inconclusive results on passion fruit effect. | Study mainly assessed sweetener effects, not passion fruit compounds. |
| Corrêa et al., 2014 | Peels | ND | 4 groups (n = 8): diet with 0%, 15%, 30% fiber and one with fiber in water. Blood glucose analysis and lipid profile. Treatment: 60 days. | Reduced glycemia, triglycerides, VLDL, insulin, leptin. Increased HDL. Best effect with 30% fiber. | Soluble fibers can be fermented and modulate gastric emptying and leptin/insulin secretion. |
| Kanakasabapathi & Gopalakrishnan, 2015a | Leaves | Aqueous | 5 groups (n = 5): control, DM, DM+Glibenclamide, DM+extract, normal+extract. Organ collection for analysis of antioxidant markers. Treatment: 30 days. | Reduced lipid peroxidation and increased antioxidant enzymes in liver and kidney. | Increased antioxidant activity (SOD, CAT and GPx); possible insulin secretion increase; pentose phosphate pathway regulation. |
| Kanakasabapathi & Gopalakrishnan, 2015b | Leaves | Aqueous | The same 5 groups, as above, with blood glucose and lipid profile, histological and enzymatic analyses. Treatment: 30 days. | Reduced blood glucose, HbA1c; increased insulin and hemoglobin; improved lipid profile; pancreatic regeneration; restored liver enzymes and glycogen. | Insulin increase; hypoglycemic and hypolipidemic effect via phytocompounds, such as flavonoids, tannins and alkaloids. |
| Kandandapani et al., 2015 | Peels and seeds | Ethanolic | 5 groups (n = 6): control, DM, DM+Glibenclamide, DM+extract (250, 500 mg/kg). Glycemic analysis, oxidative stress markers and histopathology. Treatment: 15 days. | Reduced blood glucose, increased SOD and CAT, reduced TBARS, improved weight, pancreatic protection. | Antioxidant and glycemic control activity from flavonoids, triterpenes, and steroids; glycemic control discourages the production of ROS. |
| Martins et al., 2015 | Leaves | Ethanolic (70%) | 4 groups: Control, DM, healthy + extract, DM + extract; antioxidant, phagocytic activity, protein glycation, ROS in macrophages; Treatment: 8 weeks | High antioxidant activity (DPPH, FRAP); reduced fructosamine and AGEs; restored macrophage ROS production; no significant glycemia change. | Phenolic antioxidants in extract; inhibition of protein glycation; restored ROS production in macrophages. |
| Panelli et al., 2018 | Peels | Mixed in feed (unspecified) | 2 groups (n=7): control and treated with peels. Glycemic analysis and lipid profile, oxidative stress (MDA) and antioxidant capacity. Treatment: 8 weeks. | Reduced body fat, cholesterol, TG; increased tissue antioxidant capacity; reduced MDA. | Lipid effect by soluble fibers, such as pectin, which can generate SCFAs and reduce endogenous cholesterol; carotenoids and phenolics act as antioxidants; increased mitochondrial uncoupling protein. |
| Medallon et al., 2020 | Pulp | Pulp juice | 5 groups (n = 4): control and increasing doses (1, 1.5, 2, 2.5 mL). Glycemia assessment on day 7 post-DM. | Significant glycemia reduction at 1.5, 2, 2.5 mL doses after 7 days. | Reduced blood glucose effect of pulp compounds; dose-dependent reduction. |
| Salles et al., 2020 | Leaves | Ethanolic | 80 rats, 4 groups: control, DM, healthy + extract and DM + extract. Analysis of blood glucose and lipid profile, platelet aggregation, ROS and AGEs production. Treatment: 13 weeks. | Reduced glucose, HbA1c, creatinine, total cholesterol, AGEs; inhibited platelet aggregation and ROS; kidney improvement. | Flavonoids with antioxidant, anti-AGEs, glycemic control effect; action on platelet aggregation and kidney function. |
| Holidar et al., 2021 | Pulp | Pulp juice | 6 groups (n=4): control, DM, metformin, extract (40, 50, 60 mL/kg). Analysis of lipid profile, liver proteins and MDA. Treatment: 14 days. | Reduced total cholesterol, TG, AST, ALT, MDA, best at 60 mL/kg; effect comparable to metformin. | Flavonoids (e.g., quercetin) modulate oxidative stress, inhibit lipid peroxidation; hepatoprotective; lipid reduction; adipogenesis inhibition. |
| Guan et al., 2021 | Peels | Isolated polysaccharide | 5 groups (n =5): DM+water, DM+metformin, DM+extract (50, 100, 200 mg/kg). Analysis of blood glucose, lipid profile, liver enzymes and antioxidants, SCFA, intestinal mRNA. Treatment: 5 weeks | Reduced blood glucose, increased glucose tolerance and insulin sensitivity, increased SCFAs, CAT, SOD; reduced TG, AST, ALT, hepatic lipid accumulation. | SCFA action in gut, GLP-1 increase, better insulin release; lipid peroxidation inhibition; liver repair; gut microbiota modulation. |
| Salles et al., 2021 | Leaves | Ethanolic + BuOH fraction | 80 rats (4 groups): control, DM, extract, DM + extract. Markers of renal function, lipid profile, platelet aggregation, ROS and fructosamine. Treatment: 13 weeks. | Reduced blood glucose, fructosamine, urea, total and non-HDL cholesterol; platelet aggregation and ROS reduced; classic DM symptoms improved. | Action of C-glycoside flavonoids (apigenin, luteolin, isoorientin, etc.); insulin secretion modulation; digestive enzyme inhibition; antioxidant effect. |
| Cabral et al., 2021 | Peels | Aqueous and Ethanolic | 7 groups (6 rats): DM + insulin, metformin, extracts (400/600 mg/kg) with insulin. Blood glucose and vasorelaxant evaluation. | Ex vivo vasorelaxant effect (mainly ethanolic); stronger glycemic reduction with aqueous extract. Both rich in phenols/flavonoids. | Vasorelaxation via K channel activation and phenolic compounds (isoorientin, orientin); pectin as glycemic reductor agent. |
| Galdino et al., 2022 | Peels | Aqueous | 4 groups: control, DM, DM + insulin, DM + insulin + extract. Evaluation of renal integrity markers, histology and gene expression. Treatment: 60 days | Reduced blood glucose, nephroprotective, preserved glomerular/tubular structure, reduced creatinine and urinary protein loss. | Pectin and flavonoids as glycemic control and immunomodulatory agents. Renal protection via gene expression modulation and inflammation reduction. |
| Cabral et al., 2025 | Peels | Aqueous and ethanolic | 4 groups: control, DM + insulin, DM + insulin + extract (aqueous or hydroethanolic, 400 mg/kg). Analysis of blood glucose and creatinine, histology, renal fibrosis and MPO. Treatment 60 days. | Reduced glycemia, creatinine, kidney fibrosis, MPO in kidneys/heart. No toxicity signs. Potential insulin therapy adjuvant. | Flavonoids (isoorientin, orientin, vicenin-2, vitexin) and pectin modulate glucose metabolism and insulin signaling; anti-inflammatory effect (MPO reduction). |

Legend: AGEs – Advanced Glycation End Products; ALT – Alanine Aminotransferase; AST – Aspartate Aminotransferase; CAT – Catalase; DM – Diabetes Mellitus; DPPH – 2,2-Diphenyl-1-picrylhydrazyl (free radical used in antioxidant assays); FRAP – Ferric Reducing Antioxidant Power; GPx – Glutathione Peroxidase; GTT – Glucose Tolerance Test; HbA1c – Glycated Hemoglobin; HDL – High-Density Lipoprotein; LDL – Low-Density Lipoprotein; MPO – Myeloperoxidase; mRNA – Messenger Ribonucleic Acid; ND – Not Defined or Not Described; RDI – Recommended Daily Intake; ROS – Reactive Oxygen Species; SCFAs – Short-Chain Fatty Acids; SOD – Superoxide Dismutase; TG – Triglycerides; TNF-α – Tumor Necrosis Factor; Alpha; VLDL – Very-Low-Density Lipoprotein
